# Clinical and economic impact of methicillin-resistant *Staphylococcus aureus*: a multicentre study in China

**DOI:** 10.1038/s41598-020-60825-6

**Published:** 2020-03-03

**Authors:** Xuemei Zhen, Cecilia Stålsby Lundborg, Meiling Zhang, Xueshan Sun, Yuanyuan Li, Xiaoqian Hu, Shuyan Gu, Yuxuan Gu, Jingming Wei, Hengjin Dong

**Affiliations:** 1 Center for Health Policy Studies, School of Public Health, Zhejiang University School of Medicine, Hangzhou, China; 2 School of Health Care Management, Shandong University, 44 Wenhuaxi Rd., Jinan, Shandong P.R. China; 3 Global Health-Health Systems and Policy (HSP): Medicines, focusing antibiotics, Department of Public Health Sciences, Karolinska Institutet, Stockholm, Sweden; 4 Department of Pharmacy, Tongde Hospital of Zhejiang Province, 234 Gucui Road, Hangzhou, 310012 China; 5 The Fourth Affiliated Hospital Zhejiang University School of Medicine, No. N1, Shancheng Avenue, Yiwu City, Zhejiang, China

**Keywords:** Health care economics, Health policy, Public health

## Abstract

Methicillin-resistant *Staphylococcus aureus* (MRSA) has become a serious threat to global health. In China, the proportion of *S. aureus* isolates that were MRSA was 44.6% in 2014. The clinical and economic impact of MRSA in China remains largely uninvestigated. This study aims to compare the differences in hospital costs, length of hospital stay, and hospital mortality rate between MRSA and methicillin-susceptible *S. aureus* (MSSA) colonization or infection and between MRSA cases and those without an *S. aureus* infection. A retrospective and multicentre study was conducted in four tertiary hospitals in China between 2013 and 2015. Inpatient characteristics and hospital costs were collected from electronic medical records. We conducted propensity score matching (PSM) to eliminate selection bias by balancing the potential confounding variables between the two groups. The main indicators included hospital costs, length of hospital stay, and hospital mortality rate. A total of 1,335 inpatients with MRSA, 1,397 with MSSA, and 33,606 without an *S. aureus* infection were included. PSM obtained 954 and 1,313 pairs between the MRSA and MSSA groups and between the MRSA and *S. aureus*-free groups, respectively. After PSM, MRSA colonization or infection is associated with an increased total hospital cost ranging from $3,220 to $9,606, an excess length of hospital stay of 6 days–14 days, and an attributable hospital mortality rate of 0–3.58%. Between the MRSA and MSSA groups, MRSA colonization or infection was significantly associated with a higher total hospital cost and longer length of hospital stay among survivors but not among non-survivors; however, there were no differences in the hospital mortality rate between these two groups. Between the MRSA and the *S. aureus*-free groups, MRSA colonization or infection was significantly associated with an increased total hospital cost, a prolonged length of hospital stay and a higher hospital mortality rate among both survivors and non-survivors. It is critical to quantify the clinical and economic impact of MRSA to justify resource allocation for the development of strategies to improve clinical outcomes and to reduce the economic burden.

## Introduction

*Staphylococcus aureus* is an important gram-positive bacterium in both community- and health-care-associated infections and can be resistant or susceptible to the usual antibiotics used to treat it (oxacillin or cefoxitin), namely, methicillin-resistant *S. aureus* (MRSA) or methicillin-susceptible *S. aureus* (MSSA), respectively^1,2^. MRSA has become a serious threat to global health and is responsible for a range of infections, from skin and wound infections to pneumonia and bloodstream infections. It is commonly associated with significant morbidity, hospital mortality, length of stay and economic burden^1^. In all World Health Organization (WHO) regions, the overall proportion of *S. aureus* isolates that were MRSA exceeded 20% and even exceeded 80% in some reports^3^. In China, there was a marked decrease in the proportion of MRSA from 69% in 2005 to 44.6% in 2014^4^, even though it is still higher than other common multi-drug resistant bacteria^5^.

Many studies have explored the clinical and economic impact of MRSA^6–24^. However, some studies were conducted more than a decade ago, limiting their application to the current clinical environment^6,7,9,10,15,16,18–22^. Distinct findings are presented in published literature^25,26^. It was reported that compared with patients with MSSA colonization or infection, those with MRSA are significantly associated with higher hospital costs^20,23,27,28^, higher hospital mortality rates^10,27,29^, and longer length of hospital stays^27,30^; however, in some reports, there were no differences among clinical and economic outcomes between these two groups^30,31^. Moreover, the costs for MSSA infection were higher than those for MRSA cases in one study^13^. A few studies compared the differences in clinical and economic outcomes between MRSA cases and those without an *S. aureus* infection^14,32,33^.

In China, some studies found that there were no significant differences in hospital mortality^34–36^, length of hospital stay^37^, and hospital costs^34,37^ among inpatients with MRSA and MSSA colonization or infection. Only one study explored the differences in the hospital mortality rate and length of hospital stay between MRSA cases and those without an *S. aureus* infection in China^38^. Therefore, the clinical and economic impact of MRSA remains largely uninvestigated in China. In this study, we aimed to compare the clinical and economic differences between MRSA and MSSA cases and between MRSA cases and those without an *S. aureus* infection. We also performed the analyses on both surviving and non-surviving patients.

## Materials and Methods

### Study site

This study was conducted in four tertiary hospitals in China: one in Shandong Province (site 3) and three in Zhejiang Province (site 1, site 2 and site 4). Site 1 is a combined traditional Chinese and Western medicine provincial hospital, site 2 is a general county hospital, and sites 3 and 4 are general provincial hospitals. The numbers of beds were 1,800, 1,727, 3,500 and 3,200, respectively, and the numbers of discharged patients were approximately 49,000, 76,600, 140,000 and 154,000, respectively. We chose to include these hospitals because of their relatively complete hospital information systems, which can make reliable data collection possible.

### Patients and data collection

This was a retrospective and multicentre study. First, we included 100% of inpatients in hospital sites 1–3 and 60% of inpatients in hospital site 4 between 2013 and 2015 who had clinical samples positive for MRSA colonization or infection. The control group comprised clinical samples from inpatients with MSSA colonization or infection-positive clinical samples and inpatients without an *S. aureus* infection who were hospitalized during the same study period. To avoid duplication, only patients colonized and infected with the first episode of *S. aureus* detected were included in this study. Methicillin resistance was defined as resistance to anti-staphylococcal *β*-lactam antibiotics or cephamycins (oxacillin or cefoxitin)^2^.

Inpatient characteristics were collected from electronic medical records. Variables and dates relating to demographics (age, sex, and insurance), comorbidities (disease diagnosis, and Charlson comorbidity index (CCI)^39^), hospital events (admitting service, surgical services, and dates of hospital and intensive care unit (ICU) admission/discharge), microbiological data, and clinical outcomes (death or alive during hospitalization) were extracted. Hospital costs were collected from electronic medical records as well.

### Propensity score matching

We conducted propensity score matching (PSM) using STATA to eliminate selection bias by balancing the potential confounding variables between MRSA and MSSA cases and between MRSA cases and those without an *S. aureus* infection^40,41^. We employed 1:1 nearest-neighbour matching to obtain matched pairs. MRSA was the dependent variable, and inpatient characteristics (age, sex and insurance), comorbidities, CCI, number of diagnoses, admission to the ICU, and surgery were independent variables. Then, we analysed the hospital cost, length of hospital stay, and hospital mortality rate using generated pairs.

### Indicators and data analyses

In this study, the main indicators included hospital costs, length of hospital stay, and hospital mortality rate. The total hospital cost for each inpatient’s hospitalization was calculated, including the costs paid by both inpatients and health insurance, that is, the costs of health care. It comprised medication cost (antibiotic cost), diagnostic cost, treatment cost, material cost, and other costs. All hospital costs were presented in 2015 United States (US) $ values using the 2015 consumer price index of China and the purchasing power parities^42,43^.

Hospital costs, length of hospital stay, and hospital mortality rate between MRSA and MSSA cases and between MRSA cases and those without an *S. aureus* infection were compared using Wilcoxon rank-sum test and χ^2^ test and were described using median (95% confidence interval (CI)) and proportion (95% CI) for quantitative and qualitative variables, respectively. We also calculated the differences in hospital costs, length of hospital stay, and hospital mortality rate between the two groups, which are the incremental values due to MRSA. All tests were two-tailed, and p-values < 0.05 were determined to be statistically significant. Data analyses were performed with SAS software.

### Ethical approval and informed consent

The study was approved by the institutional review board of Zhejiang University School of Public Health, who waived the need for informed consent. All inpatients data were anonymized prior to analysis.

## Results

A total of 1,335 inpatients with MRSA, 1,397 with MSSA, and 33,606 without an *S. aureus* infection were included in our study. Most of the inpatients were male (approximately 60%) and were covered by insurance (approximately 80%), and a small number of inpatients were admitted to the ICU or underwent surgery. The median ages were 74, 66, and 67 years old, the median number of diagnoses was 7, 6, and 5, and the median CCIs were 5, 4, and 4 for MRSA cases, MSSA cases, and those without an *S. aureus* infection, respectively (Tables 1–2).

**Table 1:** Characteristics of the patients with MRSA and MSSA before and after PSM.

Characteristics	Before PSM			After PSM		
	MSSA	MRSA	P-value	MSSA	MRSA	P-value
Number of inpatients, n	1397	1335		954	954	
Age in years, median (range)	66 (0–99)	74 (0–102)	<0.000	71 (1–99)	72 (0–98)	0.979
Sex male, n(%)	864 (61.85)	942 (70.56)	<0.000	645 (67.61)	637 (66.77)	0.696
Insurance, n(%)	1133 (81.10)	1049 (78.58)	0.100	769 (80.61)	771 (80.82)	0.908
Number of diagnosis, median (range)	6 (1–24)	7 (1–37)	<0.000	6 (1–24)	6 (1–22)	0.670
Charlson comorbidity index, median (range)	4 (1–39)	5 (1–27)	0.000	5 (1–34)	5 (1–27)	0.694
Admission to ICU, n(%)	148 (10.59)	352 (26.37)	<0.000	143 (14.99)	123 (12.89)	0.186
Surgery, n(%)	443 (31.71)	410 (30.71)	0.573	293 (30.71)	280 (29.35)	0.516
Myocardial infarction, n(%)	29 (2.08)	27 (2.02)	0.922	17 (1.78)	19 (1.99)	0.736
Congestive heart failure, n(%)	204 (14.60)	231 (17.30)	0.054	154 (16.14)	149 (15.62)	0.754
Peripheral vascular disease, n(%)	15 (1.07)	16 (1.20)	0.758	13 (1.36)	9 (0.94)	0.391
Cerebrovascular diseases, n(%)	652 (46.67)	720 (53.93)	<0.000	506 (53.04)	511 (53.56)	0.819
Dementia, n(%)	55 (3.94)	70 (5.24)	0.102	42 (4.40)	60 (6.29)	0.067
Chronic pulmonary disease, n(%)	210 (15.03)	289 (21.65)	<0.000	179 (18.76)	172 (18.03)	0.679
Connective tissue disease, n(%)	43 (3.08)	18 (1.35)	0.002	17 (1.78)	16 (1.68)	0.861
Mild liver disease, n(%)	59 (4.22)	41 (3.07)	0.109	35 (3.67)	35 (3.67)	1.000
Peptic ulcer disease, n(%)	28 (2.00)	53 (3.97)	0.002	26 (2.73)	24 (2.52)	0.774
Diabetes mellitus, n(%)	369 (26.41)	364 (27.27)	0.615	250 (26.21)	258 (27.04)	0.679
Diabetes mellitus with chronic complications, n(%)	49 (3.51)	26 (1.95)	0.013	23 (2.41)	22 (2.31)	0.880
Moderate to severe chronic kidney disease, n(%)	102 (7.30)	130 (9.74)	0.022	84 (8.81)	84 (8.81)	1.000
Hemiplegia, n(%)	14 (1.00)	27 (2.02)	0.028	10 (1.05)	6 (0.63)	0.315
Solid tumor without metastases, n(%)	130 (9.31)	97 (7.27)	0.054	74 (7.76)	76 (7.97)	0.865
Leukemia, n(%)	27 (1.93)	24 (1.80)	0.794	20 (2.10)	19 (1.99)	0.871
Malignant lymphoma, n(%)	18 (1.29)	17 (1.27)	0.972	15 (1.57)	15 (1.57)	1.000
Severe liver disease, n(%)	20 (1.43)	18 (1.35)	0.853	17 (1.78)	14 (1.47)	0.587
Metastatic tumor, n(%)	106 (7.59)	39 (2.92)	<0.000	39 (4.09)	39 (4.09)	1.000

**Table 2:** Characteristics of the patients with MRSA and those without an *S. aureus* infection before and after PSM.

Characteristics	Before PSM			After PSM		
	Without an *S. aureus*	MRSA	P-value	Without an *S. aureus*	MRSA	P-value
Number of inpatients, n	33606	1335		1313	1313	
Age in years, median (range)	67 (0–102)	74 (0–102)	<0.000	72 (0–102)	73 (0–102)	0.762
Sex male, n(%)	20565 (61.19)	942 (70.56)	<0.000	917 (69.84)	924 (70.37)	0.765
Insurance, n(%)	27249 (81.08)	1049 (78.58)	0.022	1015 (77.30)	1033 (78.67)	0.397
Number of diagnosis, median (range)	5 (1–25)	7 (1–37)	<0.000	7 (1–25)	7 (1–37)	0.793
Charlson comorbidity index, median (range)	4 (1–37)	5 (1–27)	<0.000	5 (1–25)	5 (1–27)	0.846
Admission to ICU, n(%)	1284 (3.82)	352 (26.37)	<0.000	337 (25.67)	333 (25.36)	0.858
Surgery, n(%)	7453 (22.18)	410 (30.71)	<0.000	420 (31.99)	407 (31.00)	0.585
Myocardial infarction, n(%)	659 (1.96)	27 (2.02)	0.874	24 (1.83)	25 (1.90)	0.885
Congestive heart failure, n(%)	6451 (19.20)	231 (17.30)	0.085	201 (15.31)	228 (17.36)	0.154
Peripheral vascular disease, n(%)	278 (0.83)	16 (1.20)	0.145	23 (1.75)	16 (1.22)	0.259
Cerebrovascular diseases, n(%)	10863 (32.32)	720 (53.93)	<0.000	709 (54.00)	701 (53.39)	0.754
Dementia, n(%)	225 (0.67)	70 (5.24)	<0.000	65 (4.95)	67 (5.10)	0.858
Chronic pulmonary disease, n(%)	11876 (35.34)	289 (21.65)	<0.000	290 (22.09)	288 (21.93)	0.925
Connective tissue disease, n(%)	1122 (3.34)	18 (1.35)	<0.000	23 (1.75)	18 (1.37)	0.431
Mild liver disease, n(%)	1604 (4.77)	41 (3.07)	0.004	45 (3.43)	41 (3.12)	0.661
Peptic ulcer disease, n(%)	986 (2.93)	53 (3.97)	0.029	46 (3.50)	52 (3.96)	0.537
Diabetes mellitus, n(%)	7045 (20.96)	364 (27.27)	<0.000	357 (27.19)	355 (27.04)	0.930
Diabetes mellitus with chronic complications, n(%)	890 (2.65)	26 (1.95)	0.116	27 (2.06)	26 (1.98)	0.890
Moderate to severe chronic kidney disease, n(%)	2507 (7.46)	130 (9.74)	0.002	127 (9.67)	126 (9.60)	0.947
Hemiplegia, n(%)	89 (0.26)	27 (2.02)	<0.000	24 91.83)	25 (1.90)	0.885
Solid tumor without metastases, n(%)	3845 (11.44)	97 (7.27)	<0.000	102 (7.77)	96 (7.31)	0.657
Leukemia, n(%)	760 (2.26)	24 (1.80)	0.262	27 (2.06)	24 (1.83)	0.671
Malignant lymphoma, n(%)	536 (1.59)	17 (1.27)	0.356	26 (1.98)	17 (1.29)	0.166
Severe liver disease, n(%)	300 (0.89)	18 (1.35)	0.086	19 (1.45)	18 (1.37)	0.868
Metastatic tumor, n(%)	1350 (4.02)	39 (2.92)	<0.000	38 (2.89)	39 (2.97)	0.908

Compared with both inpatients with MSSA and those without an *S. aureus* infection, those with MRSA were significantly associated with an older age, a higher proportion of males, a higher number of diagnoses, a higher CCI, and a higher rate of admission to the ICU (Tables 1–2). In addition, MRSA patients were more likely to have insurance coverage and undergo surgery than those without an *S. aureus* infection (Table 2). Some comorbidities between inpatients with MRSA and MSSA and between MRSA patients and those without an *S. aureus* infection were significantly different. Therefore, PSM was conducted to balance the characteristics between the two groups, and 954 and 1,313 pairs, respectively, were obtained (Tables 1–2). These pairs were subjected to analysis of the hospital costs, length of hospital stay, and hospital mortality rate. After PSM, there were no differences in baseline characteristics between the two groups.

Inpatients with MRSA were significantly associated with higher hospital costs than those with MSSA. The median differences (95% CI) in total hospital cost, antibiotic cost, medication cost, diagnostic cost, treatment cost, material cost, and other costs were $3,220 ($3,103–$3,393), $672 ($579–$723), $2,368 ($2,157–$2,633), $255 ($253–$278), $403 ($369–$413), $268 ($278–$295), and $8 ($6–$9), respectively. For surviving inpatients, the median differences in hospital costs were $3,182, $653, $2,373, $243, $429, $271, and $7, respectively. For non-surviving inpatients, there were significant differences in antibiotic cost, medication cost, and diagnostic cost, with median differences of $2,318, $3,078, and $382, respectively (Table 3).

**Table 3:** Hospital costs of patients with MRSA and MSSA after PSM for potential confounding variables.

Hospital cost ($, 2015)	MSSA			MRSA			Difference			P-value
	Median	95% CI		Median	95% CI		Median	95% CI		
**Survivor+non-survivor (%)**										
Total hospital cost	8,230	7,431	9,131	11,450	10,534	12,525	3,220	3,103	3,393	<0.000
Antibiotic cost	391	348	452	1,063	927	1,175	672	579	723	<0.000
Medication cost	3,460	3,087	3,774	5,828	5,244	6,407	2,368	2,157	2,633	<0.000
Diagnostic cost	1,321	1,242	1,400	1,577	1,495	1,678	255	253	278	<0.000
Treatment cost	1,832	1,656	2,053	2,235	2,025	2,466	403	369	413	<0.000
Materical cost	464	405	520	733	683	814	268	278	295	<0.000
Other cost	10	8	12	18	15	21	8	6	9	0.0001
**Survivor (%)**										
Total hospital cost	7,995	7,314	8,833	11,178	10,357	12,155	3,182	3,042	3,322	<0.000
Antibiotic cost	384	335	438	1,037	911	1,156	653	577	717	<0.000
Medication cost	3,305	2,961	3,657	5,678	5,068	6,262	2,373	2,107	2,604	<0.000
Diagnostic cost	1,311	1,222	1,383	1,554	1,462	1,656	243	239	273	<0.000
Treatment cost	1,794	1,627	2,045	2,223	2,020	2,467	429	393	422	<0.000
Materical cost	452	398	512	723	668	788	271	270	276	<0.000
Other cost	9	8	12	17	14	20	7	6	9	0.0001
**Non-survivor (%)**										
Total hospital cost	11,535	7,784	17,975	18,069	12,965	21,375	6,533	5,181	3,400	0.0594
Antibiotic cost	824	506	1,530	3,141	1,032	6,203	2,318	527	4,673	0.0026
Medication cost	6,754	5,217	8,135	9,832	7,113	13,268	3,078	1,896	5,133	0.0206
Dignostic cost	2,227	1,135	2,740	2,610	1,786	3,749	382	651	1,009	0.0498
Treatment cost	2,459	1,091	4,127	2,438	1,638	4,622	−21	548	496	0.2601
Materical cost	633	457	1,645	928	751	1,527	295	295	−118	0.2057
Other cost	31	18	51	30	24	75	0	6	23	0.6523

Inpatients with MRSA were significantly associated with higher hospital costs than those without an *S. aureus* infection. The median differences (95% CI) in total hospital cost, antibiotic cost, medication cost, diagnostic cost, treatment cost, material cost, and other costs were $9,606 ($9,162–$10,575), $1,112 ($1,021–$1,210), $5,516 ($5,148–$5,870), $877 ($797–$986), $1,835 ($1,677–$1,975), $671 ($621–$725), and $7 ($6–$9), respectively. For surviving inpatients, the median differences in hospital costs were $9,336, $31,065, $5,354, $817, $1,771, $654, and $6, respectively. For non-surviving inpatients, there were significant differences in hospital costs except other costs, with median differences of $13,855, $3,631, $8,047, $1,851, $2,588, and $826, respectively (Table 4).

**Table 4:** Hospital costs of patients with MRSA and those without an *S. aureus* infection after PSM for potential confounding variables.

Hospital cost ($, 2015)	Without an *S. aureus*			MRSA			Difference			P-value
	Median	95% CI		Median	95% CI		Median	95% CI		
**Survivor+non-survivor (%)**										
Total hospital cost	4,241	3,834	4,818	13,847	12,996	15,393	9,606	9,162	10,575	<0.000
Antibiotic cost	225	197	255	1,336	1,218	1,465	1,112	1,021	1,210	<0.000
Medication cost	1,619	1,443	1,805	7,136	6,591	7,675	5,516	5,148	5,870	<0.000
Diagnostic cost	988	950	1,030	1,866	1,746	2,017	877	797	986	<0.000
Treatment cost	896	816	1,005	2,731	2,493	2,980	1,835	1,677	1,975	<0.000
Materical cost	237	199	290	908	820	1,016	671	621	725	<0.000
Other cost	14	12	16	21	18	24	7	6	9	0.0125
**Survivor (%)**										
Total hospital cost	4,179	3,809	4,785	13,516	12,655	14,566	9,336	8,846	9,782	<0.000
Antibiotic cost	228	199	255	1,293	1,175	1,408	1,065	977	1,153	<0.000
Medication cost	1,606	1,422	1,770	6,960	6,430	7,485	5,354	5,008	5,715	<0.000
Diagnostic cost	988	945	1,030	1,804	1,694	1,967	817	748	937	<0.000
Treatment cost	893	810	994	2,664	2,445	2,941	1,771	1,634	1,947	<0.000
Materical cost	234	191	282	888	785	997	654	593	716	<0.000
Other cost	14	12	15	20	17	23	6	5	8	0.0413
**Non-survivor (%)**										
Total hospital cost	5,920	4,592	12,683	19,775	16,289	24,190	13,855	11,698	11,507	0.0009
Antibiotic cost	170	74	432	3,802	2,423	5,980	3,631	2,349	5,548	<0.000
Medication cost	2,945	1,954	9,331	10,992	8,945	13,905	8,047	6,991	4,574	0.0011
Dignostic cost	1,206	560	1,631	3,057	2,287	3,781	1,851	1,727	2,150	0.0003
Treatment cost	1,457	507	2,182	4,045	2,577	5,513	2,588	2,070	3,331	0.0028
Materical cost	507	230	1,589	1,333	930	1,930	826	700	341	0.0416
Other cost	32	7	56	28	24	38	−4	17	−18	0.587

The length of hospital stay was significantly longer for inpatients with MRSA than for inpatients with MSSA (27 days vs 21 days). There was a significant difference among surviving inpatients, with a median difference of 6 days, but no difference in non-surviving inpatients between the two groups (P = 0.0972). Compared with inpatients without an *S. aureus* infection, whether being a survivor or non-survivor, those with MRSA were significantly associated with a longer length of hospital stay, with median differences of 14 and 19 days, respectively (Table 5).

**Table 5:** Length of hospital stay among inpatients with MRSA and MSSA and among inpatients with MRSA and those without an *S. aureus* infection after PSM for potential confounding variables.

Inpatients	Length of hospital stay (days)								
	Survivor+non-survivor			Survivor			Non-survivor		
	Median	95% CI		Median	95% CI		Median	95% CI	
MSSA	21	20	23	21	20	23	20	12	26
MRSA	27	25	29	27	26	29	22	19	36
Difference	6	5	6	6	6	6	2	7	10
P-value	<0.000			<0.000			0.0972		
Without an *S. aureus*	14	13	14	14	13	14	8.5	4	29
MRSA	28	26	29	28	26	29	27	21	34
Difference	14	13	15	14	13	15	19	17	5
P-value	<0.000			<0.000			0.0006		

Notably, regarding the hospital mortality rate, the difference between MRSA and MSSA inpatients was not significant (P = 0.265); however, there was a significant difference between MRSA cases and those without an *S. aureus* infection (4.80% (3.64–5.96%) vs 1.22% (0.63–1.81%), respectively) (Table 6).

**Table 6:** Hospital mortality rate among inpatients with MRSA and MSSA and among inpatients with MRSA and those without an *S. aureus* infection after PSM for potential confounding variables.

Inpatients	Hospital mortality(%)			
	Median	95% CI		P-value
MSSA	3.98	2.74	5.22	**0.265**
MRSA	3.04	1.95	4.13	
Difference	−0.94	−0.79	−1.09	
Without an *S. aureus*	1.22	0.63	1.81	**<0.000**
MRSA	4.80	3.64	5.96	
Difference	3.58	3.01	4.15	

## Discussion

To the best of our knowledge, this is the first multicentre study with a large sample size exploring the clinical and economic impact of MRSA in mainland China using the PSM method. It is also the first study to quantify the clinical and economic outcome of MRSA by comparing both MSSA inpatients and those without an *S. aureus* infection. It might be that the effect of methicillin resistance on clinical and economic outcomes among inpatients with *S. aureus* is underestimated after adjusting for the confounding factors between the MRSA and MSSA groups. Compared with inpatients without an *S. aureus* infection, however, the effect of MRSA is balanced. We found that after PSM, MRSA is associated with an increased total hospital cost ranging from $3,220 to $9,606, an excess length of hospital stay of 6 days–14 days, and an attributable hospital mortality rate of 0–3.58%.

Among non-surviving inpatients, methicillin resistance does not independently increase the total hospital cost or length of hospital stay when the control group is MSSA. However, when the control group was inpatients without an *S. aureus* infection, MRSA was an independent factor for the total hospital cost or length of hospital stay among both survivors and non-survivors. Some studies reported that the difference in the total hospital cost and length of hospital stay for MRSA inpatients, compared with MSSA inpatients, are not significant^11,34,37^; however, compared with inpatients without an *S. aureus* infection, they are significant^6,21,32^, which is consistent with our findings. Some studies conducted in China reported a 7.3 times higher median total hospital cost and 14 days longer median length of hospital stay among inpatients with MRSA than those without an *S. aureus* infection after controlling for some confounding factors^11^; some studies only found significant differences between MRSA and MSSA patients using a univariate analysis, but not using a multivariate analysis^34,35^.

The length of hospital stay is a major contributing factor to hospital costs^23^. In addition to an increase in daily bed cost, the prolonged length of hospital stay may be related to more treatment and diagnostic services, which yield substantial hospital costs; therefore, we did not include length of hospital stay in PSM analyses^44^. In the case of inpatients that die during hospitalization, their illness was more likely to be associated with critical illness; thus, there is a greater possibility that they will be admitted to the ICU, undergo more surgeries, and be treated with more diagnoses and more expensive medications, and these might contribute to increased hospital costs and length of hospital stay compared to surviving inpatients, but it this can attenuate the influence of methicillin resistance. Thus, we only found a significant difference in the total hospital cost and length of hospital stay among surviving inpatients between the MRSA and MSSA groups.

The hospital mortality rate between MRSA and MSSA inpatients has long been controversial, and the results in some studies are inconsistent^12,16,17,20,33,40,45,46^. It was reported that patients with MRSA pneumonia infections had a higher mortality rate than those with MSSA (P < 0.001)^13^. In our study, we found that there were no differences in the hospital mortality rate between MRSA and MSSA inpatients; however, a significant difference existed between MRSA inpatients and those without an *S. aureus* infection, and this is consistent with some other studies conducted in China^34–36,38^. Some studies in China reported a significantly higher hospital mortality for MRSA patients than those without an *S. aureus* infection (10.94% vs 4.43%)^38^; however, there were no significant differences in hospital mortality between MRSA and MSSA patients after adjusting for confounding factors^34–36^.

A total of 67 non-surviving inpatients were included, including 29 inpatients with MRSA and 38 with MSSA. The small sample size may lack statistical power to detect significant differences; therefore, cohort studies with larger sample sizes among non-surviving inpatients in both the MRSA and MSSA groups are needed in future studies. In addition, MRSA and MSSA patients are usually empirically treated with vancomycin before the cultures are returned; however, when treated with vancomycin rather than beta-lactam agents, they showed a worse outcome for MSSA patients but a better outcome for MRSA patients^47^. Controlling the confounding factors using PSM can also be expected to bias the study towards a similar result between the MRSA and MSSA groups. Therefore, it is important to select variables in the PSM analysis. Some variables representing the severity of illness, including whether admitted to the ICU, whether undergoing surgery, complications or underlying disease, were included in the PSM analyses to decrease the influence of these variables on the main indicators; however, we excluded some variables that were directly related to hospital costs, such as length of hospital stay, length of ICU stay, and number of surgeries^44^, which is consistent with some studies^13,48^. Some caution should be taken in the interpretation of these results.

The average total hospital cost, length of hospital stay, and hospital mortality were $3,042 and $2,470, 10.1 days and 9.4 days, 0.3% and 0.4% in Zhejiang and Shandong Provinces in 2015, respectively, which were similar to the national level ($2,378, 9.6 days, and 0.4%). Therefore, we assumed that the attributable total hospital cost, length of hospital stay, and hospital mortality due to MRSA from four hospitals in China were approximate results representing the national level. We also needed to expand this study to different types of hospitals in different areas in the future.

Certain limitations are worth considering in this study. First, due to the retrospective nature of our study, it is difficult to distinguish between colonization and infection, and ascertainment bias cannot be neglected. A stronger analysis needs to be conducted only in inpatients with a clinical infection in the future. In addition, in the PSM method, we only accounted for observable covariates regardless of other unobservable covariates; thus, there may remain some hidden biases after matching. Moreover, although it is a multicentre study, the data are from tertiary hospitals only and may not apply to other types of medical institutions.

## Conclusions

Between the MRSA and MSSA groups, MRSA colonization or infection was significantly associated with a higher total hospital cost and longer length of hospital stay among survivors but not among non-survivors. There were, however, no differences in the hospital mortality rate. Between the MRSA group and the *S. aureus-free* group, MRSA colonization or infection was significantly associated with an increased total hospital cost, a prolonged length of hospital stay and a higher hospital mortality rate among both survivors and non-survivors. It is critical to quantify the clinical and economic impact of MRSA to justify resource allocation for the development of strategies to improve clinical outcomes and to reduce the economic burden.
